# The Review of Current Knowledge on Neutrophil Gelatinase-Associated Lipocalin (NGAL)

**DOI:** 10.3390/ijms241310470

**Published:** 2023-06-21

**Authors:** Katarzyna Romejko, Magdalena Markowska, Stanisław Niemczyk

**Affiliations:** Department of Internal Diseases, Nephrology and Dialysis, Military Institute of Medicine—National Research Institute, 128 Szaserów Street, 04-141 Warsaw, Poland; mkowalczyk7@wim.mil.pl (M.M.); sniemczyk@wim.mil.pl (S.N.)

**Keywords:** NGAL, acute kidney injury, chronic kidney disease, cardiovascular complications

## Abstract

Neutrophil gelatinase-associated lipocalin (NGAL) is a 25-kDa protein that is secreted mostly by immune cells such as neutrophils, macrophages, and dendritic cells. Its production is stimulated in response to inflammation. The concentrations of NGAL can be measured in plasma, urine, and biological fluids such as peritoneal effluent. NGAL is known mainly as a biomarker of acute kidney injury and is released after tubular damage and during renal regeneration processes. NGAL is also elevated in chronic kidney disease and dialysis patients. It may play a role as a predictor of the progression of renal function decreases with complications and mortality due to kidney failure. NGAL is also useful in the diagnostic processes of cardiovascular diseases. It is highly expressed in injured heart tissue and atherosclerostic plaque; its serum concentrations correlate with the severity of heart failure and coronary artery disease. NGAL increases inflammatory states and its levels rise in arterial hypertension, obesity, diabetes, and metabolic complications such as insulin resistance, and is also involved in carcinogenesis. In this review, we present the current knowledge on NGAL and its involvement in different pathologies, especially its role in renal and cardiovascular diseases.

## 1. Introduction 

**Figure 1:**
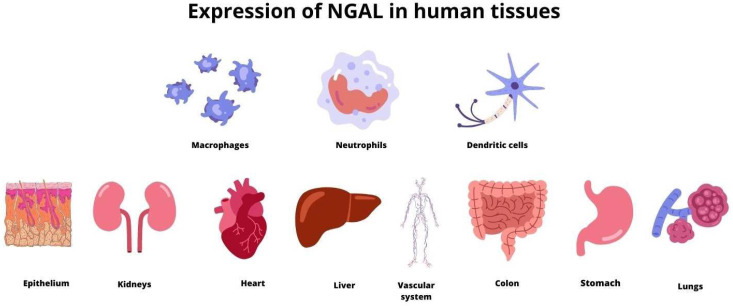
The expression of NGAL in numerous human tissues.

Neutrophil gelatinase-associated lipocalin (NGAL) is a protein that is secreted by activated neutrophils [1]. Although neutrophils are the main source of NGAL, their expression is also found in numerous human tissues, including tubular cells in the kidney, heart, lung, liver, stomach, colon, epithelial cells, macrophages, dendritic cells and adipocytes (Figure 1) [2,3,4,5,6,7,8]. NGAL is also known as lipocalin-2 and represents a family of lipocalin proteins [9]. Its structure is often described as a three-dimensional barrel [10]. It contains a ligand-binding site called calyx which allows receptors to attach to the surface of membranes and create bigger molecules [3]. Neutrophil chemoattractants, specifically N-formylated tripeptides, with leukotriene B4 and the platelet-activating factor, are the major group of ligands that connect to the binding side of NGAL [11]. There are three forms of NGAL: a 25 kDa monomer which is released by renal tubules, a 45 kDa homodimer secreted by neutrophils in an inflammatory response and a 135 kDa complex of NGAL with matrix metalloproteinase (MMP-9) [1,12,13]. The connection of NGAL to MMP-9 increases the activity of MMP-9 and protects against its degradation [14]. This results in enhanced proteolysis and the dissolution of collagen and contributes to fibrosis [15,16].

Two receptors for NGAL can be found: lipocalin-2 receptor (24p3R) and megalin receptor. The role of the lipocalin-2 receptor is multifactorial. It regulates intracellular iron concentrations. It is also expressed in cardiomyocytes with up-regulation during myocarditis and is known to be involved in smooth muscle cell proliferation, cardiac remodeling, and cardiac fibrosis [17,18]. The role of the megalin receptor is not yet well understood. Studies have shown that it binds NGAL with high affinity: much higher than other lipocalins [19]. Its expression can be observed in cardiomyocytes, kidney and ileum epithelial cells, lung ependyma, epididymis, immune cells, and numerous types of cancer cells [20,21,22]. It is involved in various cancer processes such as proliferation, migration, angiogenesis, immunotolerance, and multidrug resistance [23].

## 2. The Biological Role of NGAL

NGAL is known to be a syderophoric protein that plays a role in regulating iron activity [24]. The molecule of NGAL-containing iron interacts with receptors on the cell surface. Then, it is transported into the cell and releases iron inside [25]. NGAL that is not bound to iron also interacts with the cell surface receptors, which results in an intracellular iron transfer out of the cell [26].

### 2.1. NGAL in Infections

**Figure 2:**
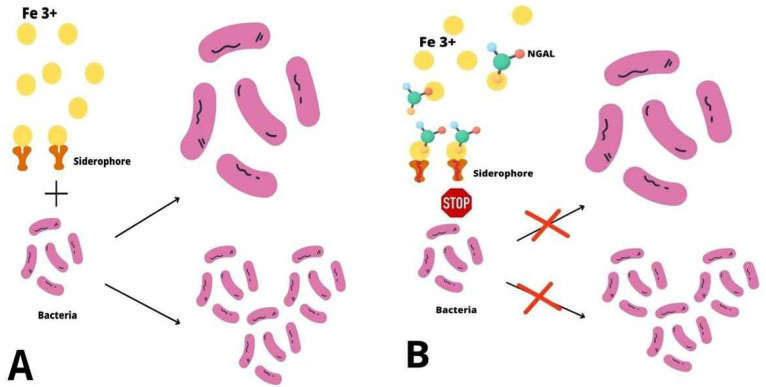
The antibacterial mechanism of NGAL. (**A**) **Bacterial iron uptake** During inflammatory processes bacteria synthesize siderophores which scavenge iron ions (Fe 3+) and transfer them into the cell, which enables bacteria to grow and proliferate. (**B**) **The role of NGAL in bacterial infections** NGAL sequestrates siderophores, prevents bacteria from obtaining iron and thus decreases their growth and multiplication.

NGAL plays a role as an acute phase protein and takes part in antibacterial immune processes. Inflammatory cytokines induce NGAL expression in neutrophils, epithelial cells, or hepatocytes [27,28,29]. The injury of epithelial cells in the intestine, stomach, liver, or lungs during infections results in an increase in plasma NGAL concentrations [30,31,32,33]. NGAL modulates iron transport as part of antibacterial immunity. During inflammatory processes, bacteria synthesize siderophores. The high affinity of siderophores for iron results in its dissociation from lactoferrin and transferrin and its transfer into the pathogen [34]. The macrophage of Toll-like receptor (TLR) stimulation up-regulates the NGAL gene and enhances NGAL synthesis. NGAL sequestrates siderophores, prevents bacteria from obtaining iron, and thus decreases bacteria growth and multiplication, as the pathogen’s ability to proliferate is often dependent on the bioavailability of iron (Figure 2) [6,34]. It has been proved that NGAL binds iron together with a metabolic product called catechol and creates complexes [35]. During experiments with mice, NGAL controlled bacterial infections by changing the iron transfer [36]. NGAL has been observed to prevent the production of siderophores by *Escherichia coli*, which could be involved in pneumonia. Moreover, NGAL expression in bronchial epithelium and alveolar type II pneumocytes is increased during respiratory infection with *Escherichia coli* [33]. NGAL also protects the respiratory system from other types of infections, such as *Stapylococcus aureus*, *Klebsiella pneumoniae*, or *Mycobacterium tuberculosis* [37,38,39]. The inflammation of mucosa during gastritis caused by *Helicobacter pylori* also increases the local expression of NGAL [31]. Additionally, NGAL up-regulates bacterial clearance from the urinary system [40]. Although the sequestration of bacterial siderophores is the major bacteriostatic function of NGAL, it is also involved in the activation and transformation of T- cells towards the Th1 type [41]. It has been found that the function of neutrophils is impaired in the state of an NGAL deficit. This may result in the dysfunction of chemotaxis and the adhesion and migration of inflammatory cells [42]. Studies have shown that patients with decreased NGAL levels are more prone to various infections [6].

NGAL concentrations are increased in sepsis and correlate with inflammatory parameters such as interleukin-6 (IL-6), interleukin-10 (IL-10), vascular cellular adhesion molecule-1 (VCAM-1), intercellular adhesion molecule 1 (ICAM-1), tumor necrosis factor-alpha (TNF-alpha), the C-reactive protein (CRP) and leukocytes account [43,44,45]. Additionally, plasma NGAL levels are higher in patients with septic shock and sepsis-related organ failure compared to those with a milder course of sepsis. Lentini found an association between the severity of a systemic inflammatory response and a higher plasma NGAL level in patients with sepsis and acute kidney injury [46]. However, further studies are necessary to assess if the risk of mortality is also higher in individuals with elevated plasma NGAL concentrations [43,47,48].

NGAL is also involved in fungal and viral infections. Despite the fact that the exact role of NGAL in fungal infection is not yet well known, a better expression of the NGAL gene was found in candidiasis [49]. The induction of NGAL gene expression has been documented in epithelial cells in patients with infections caused by *human papillomavirus* and *rotavirus* [50,51]. In children with dehydration caused by *rotavirus* infections, the NGAL level can be an early indicator of renal impairment [52]. By contrast, in patients with a *human immunodeficiency virus (HIV)*, the serum NGAL level is low due to the decreased number and impaired function of neutrophils [53].

### 2.2. NGAL in Metabolic Complications

NGAL concentrations rise in many pathological states [51,52]. NGAL is expressed in numerous types of tissues, and its concentrations increase during injury. Inflammatory cytokines induce NGAL expression in neutrophils, epithelial cells, or hepatocytes [53,54,55]. Adipose tissue is also the source of NGAL, NGAL concentrations are higher in obesity, diabetes mellitus type 2, and nonalcoholic fatty liver disease [54,55]. There are studies that have proved that the estimation of NGAL concentrations in serum could be useful in predicting the metabolic complications caused by obesity [55,56]. NGAL is an independent risk factor for insulin resistance, systolic blood pressure, and lipid metabolism disorders [56,57,58,59,60]. The role of NGAL in the pathogenesis of obesity is not yet well understood. However, it is associated with the regulation of a proliferator-activated receptor gamma (PPAR gamma)—a molecule that participates in adipogenesis and lipogenesis [61]. A study by Zhang found that obese mice had high levels of NGAL mRNA, which induced PPARgamma [61]. The anti-inflammatory function of NGAL in adipose tissue is also possible by inhibiting nuclear factor κB (NFκB) activity, which antagonizes the effect of TNF-alpha on local inflammation in fat, and increases IL-6 and monocyte chemoattractant protein-1 (MCP-1) production or inhibits the secretion of leptin and adiponectin [61,62]. Numerous studies have shown a significant association between NGAL and the activity of 12-lipoxygenase and TNF-alpha in adipose tissue. The function of these molecules is crucial to the development of insulin resistance. It has been proved that NGAL stimulates its expression and that the deficit of NGAL protects from the progress of glucose metabolism disorders [56,57]. It was also documented that the synthesis of NGAL was stimulated by hyperglycemia [63]. An NGAL urinary excretion is a marker of diabetic nephropathy and is detected in urine early as a result of high serum glucose concentrations, even before kidney injury [64,65]. Urine NGAL concentrations correlate with the stage of renal damage in diabetic nephropathy [66]. Moreover, Hafez proved in their study that the level of urine NGAL in children with diabetes could be associated with an albumin-to-creatinine ratio, the duration of diabetes, glycated hemoglobin concentrations, and dyslipidemia [67].

### 2.3. NGAL in Carcinogenesis

NGAL is also known as a growth factor. It stimulates the proliferation and differentiation of epithelial cells [68]. Due to this fact, the role of NGAL has been assessed in different types of cancer. In the experiment with mouse mammary tumor cells, Hanai discovered that NGAL participated in the conversion of 4T1-Ras-transformed mesenchymal tumor cells to an epithelial phenotype. It increased E-cadherin expression, which resulted in a rise in cell invasiveness, tumor growth, and lung metastases [69]. Human studies have also demonstrated that NGAL promotes breast cancer progression through the induction of mesenchymal markers such as vimentin or fibronectin [70,71]. The deficit of NGAL also impaired the migration of tumor cells [72]. Conflicting data from this research might be caused by the activation of Ras oncogen in one setting but not in another [73]. In thyroid carcinoma, NGAL is responsible for the existence of tumor cells by regulating NFκB activity, which is connected with iron homeostasis [74]. The pro-metastatic role of the NGAL/MMP-9 complex in aggressive thyroid carcinomas was also considered [75]. In gastric cancer, the detection of this complex has been associated with a worse prognosis [76,77]. The increased activity of NGAL/MMP-9 accelerates the migration and invasion of malignant cells and promotes the metastasis of esophageal squamous cell carcinoma and prostate cancer [78,79,80,81]. Numerous studies have shown that NGAL has the ability to bind with MMP-9 and subsequently scavenge iron into cancer cells, thereby increasing the aggressiveness of the disease. Hopefully, NGAL could even be a biomarker of malignancy and the aim of anticancer therapies in the future, but further research is necessary [14,82,83,84,85].

## 3. NGAL in Kidney Diseases

### 3.1. NGAL in Acute Kidney Injury

In 2003 Mishra proposed that NGAL could be an early biomarker of acute kidney injury (AKI), initially in experimental and then clinical studies [86,87]. They found that in a mouse model of renal ischemia injury, NGAL was a very sensitive marker of ischemic AKI, and its urine concentrations were associated with the severity and duration of ischemia [86]. Many clinical studies have followed these observations [88,89,90]. Numerous reports have proved that NGAL is synthesized in kidney tissue following several mechanisms of kidney injury, such as ischemic, nephrotoxic, or septic [43,87,91,92,93,94]. NGAL can be detected in plasma within two hours of AKI, with a concentration peak after 6 h. Increased serum NGAL levels were observed for approximately five days after AKI before they decreased [95]. AKI also resulted in the elevation of urine NGAL levels. Elevated serum and urine NGAL, due to AKI, were observed 24 h earlier than the increase in creatinine [87,96]. There was no superiority of plasma NGAL over urine NGAL and urine NGAL over plasma NGAL in AKI, and these can be used according to laboratory preference [97].

It is nowadays known that NGAL is a marker of renal tubular damage as it is released from the distal tube [98]. The molecule is filtered through the glomerular membrane and is reabsorbed in the proximal tubule of the kidney. The NGAL observed in urine is caused by proximal tubular damage or originates from its up-regulated synthesis in the distal part of the nephron, especially in the ascending limb and Henle’s loop, and in the collecting duct [99]. The expression of NGAL in the regenerating tubular epithelial cells increases significantly after kidney injury [68,100]. Thus, NGAL in urine during AKI often originates from an impaired up-regulation in the proximal tubule segments and also from its intensified synthesis and secretion in the distal parts of the nephron. Increased NGAL synthesis in tubular epithelial cells, even in the early stages of AKI, often results from kidney regenerative processes. NGAL provides iron intracellular availability and, thus, promotes kidney regeneration. Increased plasma NGAL levels during AKI are multifactorial. One of the sources of NGAL in the serum is the activation process of neutrophils and monocytes during the acute phase of the reaction [101]. Additionally, the synthesis of NGAL in the liver and lungs during AKI is significantly elevated [99]. Moreover, a decrease in renal function results in the accumulation of NGAL in plasma, and an increase in its serum concentrations could be observed [98].

**Figure 3:**
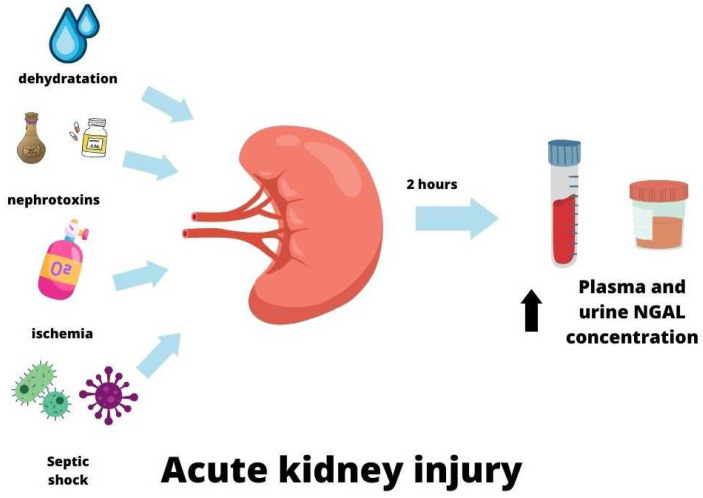
NGAL in acute kidney injury.

In conclusion, NGAL can be treated as a biomarker of acute kidney injury. However, its usefulness is limited in some diseases, especially when systemic inflammation occurs [102]. Some studies have indicated that NGAL levels may be preferable to serum creatinine concentrations for AKI prediction when measured at the same time [103,104]. The mechanisms of increased NGAL are presented in Table 1 and Figure 3.

### 3.2. NGAL in Chronic Kidney Disease

Patients with kidney function decrease have elevated serum NGAL concentrations compared to healthy controls, but the differences in serum NGAL levels between AKI and chronic kidney disease (CKD) patients are controversial. Some studies have proved that patients with AKI have higher plasma NGAL levels than patients with CKD. Other studies, however, have shown the opposite results [105,106]. The study of Gharishvandi found that NGAL was superior to cystatin C and creatinine in detecting a kidney function decrease in the early stages of CKD and in predicting its progression [107,108]. It has also been found that in patients with CKD, NGAL concentrations were negatively correlated with the eGFR value, which reflected the severity of kidney damage [99,108,109,110]. It is also known that NGAL can predict mortality in kidney function decreases [111]. The meta-analysis of Zhou revealed that NGAL could be an independent risk predictor of end-stage renal disease and mortality [112].

NGAL is secreted from injured kidney cells, and by taking part in proliferative processes and apoptosis, it is responsible for controlling tubular cells [113,114]. It mediates epidermal growth factor receptor (EGFR) signaling, whose activation stimulates hypoxia-inducible factor (HIF-1α) and finally enhances proliferation, cytogenesis, renal damage, and CKD progression [115,116,117]. Xiang found that NGAL can be associated with iron storage in CKD patients. They observed that NGAL was conversely correlated with hemoglobin, hematocrit, mean corpuscular volume (MCV), mean corpuscular hemoglobin (MCH), serum iron, and transferrin saturation (TSAT) [118]. Further research is needed, but plasma NGAL can hopefully become a more useful marker than serum ferritin for determining the iron status in CKD, including patients who require renal replacement therapy [119,120,121,122].

NGAL concentrations are elevated in different types of CKD. Its diagnostic value has been tested in the differentiation of primary inflammatory and non-inflammatory etiologies of CKD [123]. Ding compared the urinary levels of NGAL, creatinine, and N-acetyl-beta-D-glucosaminidase (NAG) in 40 healthy individuals and 70 patients with IgA nephropathy (IgAN) [124]. According to their findings, NGAL was the most sensitive marker of renal tubular injury in IgAN. This hypothesis was confirmed by Rhee, who also emphasized the prognostic value of NGAL in IgAN [125]. NGAL is also one of the best diagnostic markers in patients with SLE [126,127,128]. Some studies have found that NGAL acts as a nephroprotective agent. It probably modulates apoptosis in tubular cells and macrophages; however, additional studies are needed to clarify this mechanism [127,129]. Serum NGAL concentrations increase in diabetic nephropathy and correlate with the severity of kidney damage. Since an increase in the NGAL level of diabetic nephropathy occurs in the early stage of kidney injury, even in patients with normoalbuminuria, NGAL can play a role as a predictor of renal failure [66]. The role of NGAL in patients with autosomal dominant policystic kidney disease (ADPKD) has also been studied [130,131,132]. NGAL concentrations elevate with the impairment of renal function and with an increase in the number of cysts. Thus, NGAL has been suggested to be involved in the cyst growth process in ADPKD [130]. However, its role in ADPKD is not clear [133]. Some reports have shown that NGAL is able to inhibit cyst enlargement [134].

### 3.3. NGAL and Dialysis

NGAL has been found to be an independent predictor of CKD progression [108]. HD patients with residual renal function have significantly lower serum NGAL concentrations compared to anuric individuals. Thus, NGAL levels can also reflect the degree of kidney function decreases in end-stage renal disease (ESRD). In the group of patients treated with HD, apart from renal dysfunction, low-grade inflammation, which is more intensified in anuric individuals, can contribute to increased serum NGAL levels [135]. Arteriovenous fistula (AVF) is the preferred vascular access for HD over an arterious graft (AVG) and a central venous permanent catheter (CVPC) due to a lower risk of infection episodes. Serum NGAL was found to be higher in CVPC patients compared to those with AVF and AVG. Moreover, its plasma concentrations correlated with the duration of the catheter and also with increased inflammatory parameters such as the high sensitivity of CRP (hs-CRP), IL-6, TNF-alpha, and ferritin. Thus, NGAL can act as an inflammatory marker in HD patients, especially when hemodialised with the use of CVPC, especially in those who are mostly susceptible to infections. The same study found an inverse relationship between serum NGAL and albumin in HD patients which could indicate the role of NGAL in the poor nutritional status of this group [45]. Imamaki also revealed that NGAL is related to malnutrition in patients treated with HD. They found a significant relationship between plasma NGAL and markers of nutrition, such as muscle mass and protein intake [136]. Apart from inflammation and residual renal function in ESRD, serum NGAL concentrations were also associated with iron deficiency anemia (IDA), which is higher in HD individuals with lower tranferrin saturation [137]. Very similar results were observed in the study of Aghsaeifard, which included 47 HD participants. The serum NGAL concentrations correlated significantly with IDA. They also found a negative relationship between serum NGAL and nutritional parameters such as albumin and total cholesterol, which could suggest the role of NGAL in the development of malnutrition in CKD patients treated with HD [138]. Jie investigated the association between NGAL and mineral bone disorders in HD patients, which are common complications of CKD. They found that plasma NGAL levels were associated with serum phosphatase, calcium, and phosphate, which suggests that NGAL could contribute to mineral bone disorders in HD individuals [139]. The study of Yigit with 61 HD participants found a positive relationship between serum NGAL and parathormone (PTH) in patients with severe hyperparathyroidism. They also revealed a significantly negative relationship between NGAL and hemoglobin in the group of HD patients with severe hyperparathyroidism [140].

NGAL reflects the status of the peritoneal membrane in peritoneal dialysis (PD) patients. Peritonitis, which is one of the complications of peritoneal dialysis, is associated with an increased risk of death, hospitalization rate, and peritoneal membrane failure. The diagnosis of peritonitis is made based on numerous clinical symptoms, including abdominal pain, nausea, vomiting, diarrhea, constipation, or a cloudy effluent. Laboratory measurements have revealed increased inflammatory parameters and a positive peritoneal fluid culture. However, there is often a lack of specific markers for peritonitis which could be useful in early diagnosis and enable the implementation of treatment in the initial stage of peritonitis. As NGAL was found in a peritoneal dialysis effluent, some studies have suggested that it may be considered a marker of perotonitis. The report of Virzì, which examined 27 episodes of peritonitis in 22 PD patients, found a positive relationship between NGAL concentrations and the WBC count in peritoneal dialysis effluent. This association was observed at each stage of peritonitis, with an increase in WBC and NGAL in the peritoneal dialysis effluent during the course of the disease, alongside their decrease in the recovery process. This study confirmed that peritoneal NGAL could be a reliable marker of peritonitis; moreover, it may be used in monitoring the course of the disease [141]. Previous studies have shown similar results. The report of Martino included 182 PD patients, among which 80 episodes of peritonitis were observed. They also found that NGAL in peritoneal dialysis effluent could be a marker of peritonitis episodes [142]. Lacquaniti proved that changes in peritoneal fluid NGAL concentrations in patients with peritonitis could play a diagnostic role in the process of treatment. They observed that the levels of NGAL in the peritoneal dialysis effluent fell at least 24 h earlier than the peritoneal WBC count [143].

### 3.4. NGAL in Kidney Transplantation

Numerous studies found that NGAL could be a marker of graft functions after kidney transplantation [144,145,146]. Higher plasma NGAL concentrations were observed before acute graft rejection [147]. Since serum NGAL levels were elevated in numerous pathologies, which could lead to graft dysfunction, increased NGAL concentrations and graft failure could be diagnosed by performing a biopsy and histopathology examination [148]. Additionally, the changes in NGAL levels could also indicate how fast the graft was going to assume its function [149,150]. Urinary NGAL concentrations in patients after kidney transplants with good graft function did not differ from healthy individuals [148,151].

## 4. The Role of NGAL in Cardiovascular Diseases

NGAL concentrations increased in patients with cardiovascular complications. NGAL could be expressed in heart tissue and in atherosclerotic plaques. Its plasma levels were elevated in coronary artery disease and also in acute and chronic heart failure [10,152,153,154,155]. Increased serum NGAL levels in patients with cardiovascular complications could be associated with renal dysfunction; however, numerous studies also found high NGAL concentrations in patients with cardiovascular events without kidney injury [156,157].

### 4.1. NGAL in Atheroslerosis

Inflammatory processes are often involved in the development of atherosclerosis from the onset of endothelial dysfunction through the formation of atherosclerotic plaque, which finally leads to plaque rupture with the formation of occlusive thrombus and the occurrence of acute coronary syndrome [158]. Activated neutrophils were found in atherosclerotic plaque [159]. In response to TNF-alpha, bone marrow-derived macrophages release NGAL and their stimulation by NGAL causes the up-regulation of M1 macrophage markers, the expression of scavenger receptor class A-1, and their conversion into foam cells [160]. NGAL may increase proteolytic activity inside the atherosclerotic plaque, which contributes to lower atherosclerotic plaque stability and, in consequence, also leads to cardiovascular events. It was assumed that the connection of NGAL with MMP-9, which potentiated the proteolytic activity of MMP-9, could result in plaque instability. The activation of MMP-9 is thought to be one of the mechanisms responsible for the development of inflammation and atheroslerosis [153,161]. The concentrations of the NGAL/MMP-9 complex were increased in the atherosclerotic plaque with central necrosis or hematoma, which are the states that make the atherosclerotic plaque more vulnerable to rupture [162]. Boekhorst found that the NGAL/MMP-9 complex was highly expressed in human atherosclerotic plaque and assumed that NGAL could be a novel tool for detecting patients with high-risk atherosclerotic plaques [163]. Patients with symptomatic carotid atherosclerosis had higher plasma NGAL concentrations compared to asymptomatic individuals. In addition, individuals with vulnerable plaque had higher levels of serum NGAL [164]. The study of Kafka, which included 140 patients with coronary artery disease, showed that serum NGAL concentrations increased with the severity of coronary artery disease—stable angina, unstable angina, non-ST-segment elevation myocardial infarction (NSTEMI), and ST-segment elevation myocardial infarction (STEMI)—with the highest value in STEMI patients [165]. This correlation has also been confirmed in other studies [166,167]. Moreover, Sahinarslan found that patients with higher serum NGAL concentrations suffered from an increased incidence of acute myocardial infarction compared to individuals with stable coronary artery disease who had lower plasma NGAL levels [168]. The study of Lialso also revealed that patients with STEMI had significantly higher NGAL levels compared to those with stable angina or the control subjects [169]. However, even in the case of stable coronary artery disease, the concentrations of the NGAL/MMP-9 complex were higher than in healthy individuals [170]. The study of Chen, which included 177 participants with major adverse cardiovascular and cerebrovascular events (MACCE), proved that increased serum NGAL concentrations were significantly associated with the risk of MACCE [171]. Advanced atherosclerosis could be related to arterial stiffness and is known to be associated with worse outcomes [172]. In the study by Soylu, which included 101 patients, plasma NGAL concentrations negatively correlated with an aortic strain and distensibility but were positively associated with aortic stiffness [173].

### 4.2. NGAL in Myocardial Infarction

Plaque NGAL concentrations increased in patients with acute myocardial infarction (MI). Inflammation plays a crucial role in the ischaemia-reperfusion injury of the heart [174]. Neutrophils infiltrate the infarction area and initiate inflammatory processes which enable a reduction in the damaged area; however, on the other hand, their excessive infiltration may be unfavorable for myocardial healing. NGAL is one of the numerous glycoproteins that are secreted by neutrophils during myocardial infarction, and its concentrations in the left ventricle after one week of myocardial infarction are significantly elevated [175]. Increased serum NGAL levels in patients with MI are associated with negative outcomes [176]. The study of Avci, which included 235 patients with STEMI, showed that plasma NGAL concentrations were significantly higher in patients who died compared to participants who survived. Additionally, patients with a decreased ejection fraction (EF) after MI had elevated serum NGAL levels, unlike participants with preserved EF [177]. Echocardiographic analysis revealed that myocardial function after ischemic injury was better in individuals with lower plasma NGAL concentrations [178]. Additionally, the use of anti-NGAL antibodies in a study with mice resulted in a reduced number of macrophages and neutrophils in the ischemic zone and the limitation of cardiac lesions [174]. The report of Karetnikova showed that patients with increased serum NGAL concentrations after 12 days of STEMI had a higher incidence of adverse outcomes such as recurrent myocardial infarction, early post-infarction angina, an acute cerebrovascular event, or death [179]. Additionally, the meta-analysis of Fan found that patients with STEMI and higher plasma NGAL concentrations had a 47% to 52% greater risk of all-cause mortality and major adverse cardiovascular events (MACE). They assumed that the value of plasma NGAL levels in patients with STEMI could be a tool to stratify patients with STEMI in clinical practice [180]. NGAL is an independent predictor of both MACE frequency and mortality after STEMI [181,182].

### 4.3. NGAL in Heart Failure

The inflammation and degradation of the matrix are the processes involved in the pathogenesis of heart failure (HF). It has been found that NGAL is involved in cardiac remodeling and, therefore, could be considered as biomarker for HF [161,183,184,185]. The NGAL/MMP-9 complex increases MMP-9’s enzymatic activity and intensifies cardiac remodeling. NGAL also participates in immune cell migration processes, which acts as a growth factor and promotes the proliferation and differentiation of vascular smooth muscle cells or cardiac fibroblasts [10]. Serum NGAL levels were increased in patients with acute and chronic heart failure due to myocardial infarction [186]. It has been proved in both clinical and experimental studies that the increase in plasma NGAL is the result of myocardial injury, which confirms that immune processes participate in the pathogenesis of HF [186]. Patients with chronic HF and increased serum NGAL serum concentrations have a higher mortality rate; therefore, NGAL may be a prognostic marker of survival in this group [187]. Serum plasma NGAL levels in individuals with chronic HF have been observed to be negatively correlated with EF [188]. Additionally, patients with chronic HF have higher serum NGAL concentrations compared to healthy subjects. Moreover, plasma NGAL levels increased with the severity of HF, with the highest in class IV of the NYHA (New York Heart Association) classification [187].

NGAL may also be considered a biomarker of acute HF. Individuals with acute HF and increased serum NGAL concentrations experienced a higher mortality rate, as well as a higher incidence of cardiovascular complications and hospital readmission [189]. Therefore, serum NGAL levels could help to stratify patients with acute HF. Plasma NGAL concentrations at the time of discharge from the hospital were found to be a predictor of 30-day outcomes in patients with acute HF [190].

### 4.4. NGAL in Cardiorenal Syndrome

The estimation of NGAL concentrations could predict AKI during hospitalization due to HF [189]. NGAL, as a marker of renal dysfunction, is useful in identifying HF patients in the early stages of kidney function decreases who are at high risk of developing cardiorenal syndrome (CRS). CRS is defined as a multi-organ disease, which is caused by an interaction between the heart and kidney when the acute or chronic dysfunction of one organ leads to acute or chronic dysfunction in the other. There are five types of cardiorenal syndrome that are based on primary organ failure, causing the derangements of the second one—cardiorenal or renocardiac, acute, or chronic. Type 5 cardiorenal syndrome can be diagnosed if systemic diseases such as sepsis, diabetes mellitus, or amyloidosis lead simultaneously to kidney and heart dysfunctions [191]. Among numerous classical biomarkers of acute kidney failure, such as serum creatinine, eGFR, albuminuria, and cystatin C, NGAL was found to be one of the earliest markers of renal injury. The study by Alvelos reported that the determination of serum NGAL concentrations in patients with acute HF and preserved renal function at admission to the hospital may be a helpful tool in diagnosing an early stage of type 1 CRS [192]. Numerous recent studies have confirmed that NGAL might be a predictive marker of acute kidney injury due to heart failure. The report by Nasonova proved that increased urine NGAL concentrations were prognostic markers of kidney function decreases in patients with acute decompensation of chronic heart failure [193]. The study of Dankova also proved elevated urine NGAL levels at admission to the hospital due to acute HF and predicted the following development of AKI [194]. Moreover, the report by Thai, which included 139 patients with acute heart failure, revealed that increased plasma NGAL concentrations along with elevated creatinine levels could predict the development of CRS 1 [195]. Since the rise in plasma NGAL exceeded an increase in serum creatinine, this also played a predictive role in the onset of acute kidney failure and thus allowed us to implement preventive therapeutic procedures [196]. As NGAL alone may be an independent predictive marker of CRS, the combination of serum NGAL with NT-proBNP is helpful in the early diagnosis of CRS 1 [197]. In an experimental model of chronic heart failure, an increase in NGAL concentrations could aggravate heart and renal dysfunctions by elevating the enzymatic activity of MMP-9 and intensifying extracellular matrix degradation. Thus, NGAL may also take part in the pathogenesis of type 2 CRS [198].

### 4.5. NGAL in Arrhythmias

As NGAL participates in cardiac remodeling, it could also be hypothesized that it is also involved in the occurrence of atrial fibrillation (AF) episodes [199]. The maintenance of sinus rhythm after an episode of atrial fibrillation and electrical cardioversion depends on various factors, e.g., the left atrium size, age, or the longevity of atrial fibrillation. The study of Mlodawska found that plasma concentrations of the NGAL/MMP complex could predict AF recurrence after successful electrical cardioversion in obese patients. The increased concentrations of serum NGAL/MMP levels were positively associated with the recurrence of AF [200].

### 4.6. NGAL in Hypertension

Hypertensive patients had higher plasma NGAL concentrations compared to normotensive individuals [201]. Primary hyperaldosteronism is one of the main causes of secondary hypertension. It has been proved that NGAL is involved in the development of hypertension, which is induced by increased aldosterone concentrations. The exact mechanism is still not entirely known. It is assumed that it may be associated with the activation of immune cells [202]. After the stimulation of dendritic cells and macrophages, which took place during aldosterone excess, the synthesis of NGAL increased and led to the fibrosis of blood vessels and the development of hypertension [203,204,205]. Moreover, as NGAL is an important biomarker of renal dysfunction, it may be helpful to detect kidney damage in the early stages of kidney failure in hypertensive individuals [206]. Serum NGAL concentrations correlate with ambulatory blood pressure values; thus, it is possible to diagnose a group of patients with a higher risk of cardiovascular diseases and mortality due to increased plasma NGAL [207].

### 4.7. NGAL in Cardiac Surgery

There are numerous studies that have shown that plasma and urine NGAL concentrations increase in patients undergoing cardiac surgery [208,209]. Elevated serum NGAL is associated with acute kidney injury, which is a frequent complication of heart surgery. Thus, NGAL is a marker of renal insufficiency in patients undergoing cardiac surgery [210,211].

### 4.8. NGAL in Abdominal Aortic Aneurysm

NGAL is also considered a promising marker for pathogenesis and the progression of abdominal aortic aneurysms (AAA) [212]. Polymorphonuclear neutrophils isolated from patients with AAA were able to secrete more NGAL. In a mouse model with NGAL depletion or anti-NGAL treatment, it was proved that the formation of AAA was limited, possibly due to the depletion of neutrophils [213]. Ramos-Mozo discovered that luminal thrombus released large amounts of NGAL in comparison with abluminal AAA thrombus, the AAA wall, and healthy aortic media [214].

## 5. NGAL in Biological Fluids and Additional Functions of NGAL

NGAL is also present in biological fluids. Agrawi studied NGAL expression in the saliva and tears of patients with primary Sjögren’s syndrome (pSS) and found that NGAL was up-regulated in both of them [215]. The continuation of this study was the research, which for the first time, explored NGAL expression at the site of inflammation in the pSS target organ. The results of this report were very promising. They found that the expression of NGAL in the acinar and ductal epithelium of the salivatory gland of pSS was significantly higher compared to patients without pSS [216]. Thus, NGAL may hopefully become, in the future, a promising marker for diagnostic procedures of pSS and monitoring the disease in the future.

NGAL has also been observed in human breast milk postpartum. Metallinou investigated the changes in NGAL concentrations in human breast milk in normal pregnancies and pregnancies that developed insulin-dependent gestational diabetes mellitus (iGDM). They found that the concentrations of NGAL were the highest on the first day of colostrum, with a fall after two days in both groups. The milk levels of the NGAL/MMP-9 complex also decreased in normal pregnancies as well as iGDM. However, in the second sample, the concentrations of the NGAL/MMP-9 complex were significantly higher in diabetic women. The physiological role of milk NGAL needs further study. The researchers of NGAL in human milk speculate that if NGAL plays a bacteriostatic role and takes part in mucosal healing, it may exert a protective influence on the neonatal gastrointestinal tract [217].

NGAL concentration changes in cerebrospinal fluid (CSF) were also observed in patients with cognitive impairment. Naudé found that concentrations of NGAL in CSF were significantly lower in participants with mild cognitive impairment compared to patients with subjective cognitive decline. Moreover, lower CSF NGAL concentrations predicted a conversion from mild cognitive impairment to Alzheimer’s disease. Therefore, CSF NGAL may possibly become a marker of cognitive impairment worsening [218]. The study of Wu found that peripheral blood concentrations of NGAL were higher in mild cognitive impairments and Alzheimer’s disease compared to the healthy controls, which allowed them to assume that increased neutrophil activation expressed in elevated blood NGAL levels could take part in cognitive impairments due to Alzheimer’s disease [219]. Patients with central nervous system (CNS) infections had significantly higher NGAL concentrations in CSF compared to healthy individuals. Moreover, patients with electroencephalography (EEG) abnormalities also revealed higher CSF NGAL levels. Thus, increased NGAL CSF could suggest EEG abnormalities in patients with CNS infections [220]. The CSF NGAL concentrations of patients with a subarachnoid hemorrhage (SAH) increased significantly up to the fifth day after SAH, and then they began to decrease. As NGAL was released by neutrophils, this indicated their important role in inflammatory processes in the brain after SAH [221].

NGAL was also estimated as a marker of prosthetic joint infection. NGAL in synovial fluid was found to be a marker of inflammatory processes in the prosthatic joint with promising diagnostic performance [222]. Very similar results have also been observed in recent numerous studies [223,224,225].

The complex of NGAL/MMP-9 was also evaluated in the urine and CSF of pediatric patients with moyamoya disease. The study of Sesen provided the hypothesis that the NGAL/MMP-9 complex may be useful as a predictor of the presence of moyamoya disease and may enable the monitoring of the process of treatment [226].

The expression of NGAL in the lung tissue of patients with chronic obstructive pulmonary disease (COPD) is higher compared to the healthy controls, which is the result of an increased number of neutrophils in COPD airways. NGAL may participate in the progression of COPD by enhancing airway remodeling. NGAL intensifies the proliferation and migration of human bronchial smooth muscle cells. NGAL also intensifies the epithelial-mesenchymal transition through the downregulation of E-cadherin expression and up-regulation of the expression of α-smooth muscle actin, which results in increased organ fibrosis. Wang suggested that NGAL could become a target for the novel treatment of airway remodeling and obstruction in COPD patients [227].

NGAL may also act as a novel marker in ventilator-associated lung injury. Chen conducted an experimental model of ventilator-associated lung injury. NGAL expression in bronchoalveolar lavage fluid was elevated in all methods of mechanical ventilation treatment, which increased with the degree of lung injury and was highest in the most severe lung damage [228].

The concentrations of NGAL may be measured in pleural fluid. The report by Wu found a positive correlation between serum and pleural fluid NGAL in complicated parapneumonic effusion (CPPE) and empyema; hence, serum NGAL may be a biological marker of these two pathological states [229]. NGAL may also be useful in discriminating pleural and peritoneal malignant effusion and tuberculous pleural effusion [230].

NGAL is also present in vaginal fluid. Beghini studied the relationship between NGAL and the bacterial vaginosis of reproductive-age women. They stated that the derangements of vaginal NGAL concentrations could be due to a decrease in Lactobacilli. This may result in an overgrowth of anaerobic or facultative Gram-negative bacteria, which are usually associated with bacterial vaginosis [231]. The report of Beghini also found that the concentrations of NGAL in the vaginal fluid were higher in women with vaginal inflammation [232].

The levels of NGAL were also assessed in amniotic fluid with significantly increased values in the microbial invasion of the amniotic cavity and histological chorioamnionitis in women with the pre-labor rupture of membranes [233].

## 6. Summary

**Figure 4:**
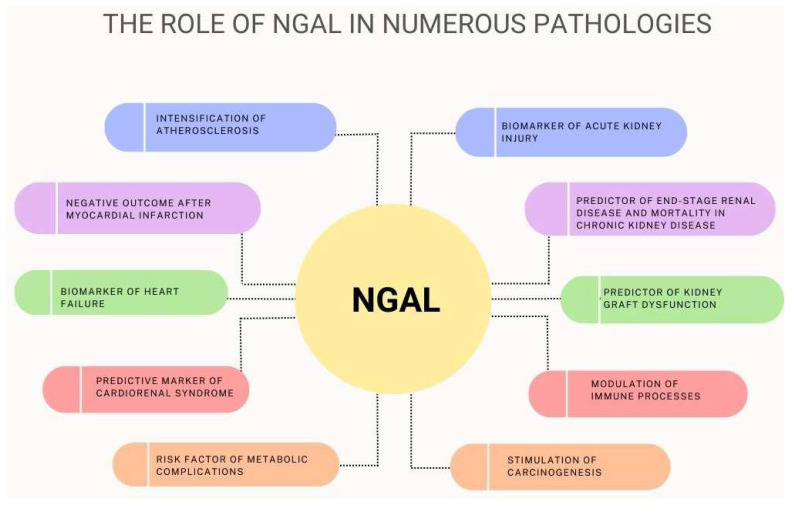
The role of NGAL in numerous pathological states.

NGAL is a protein that is secreted mainly by neutrophils, with its expression found in a wide variety of human tissues such as the kidney, heart, lung, liver, stomach, and colon. NGAL is an acute-phase protein and plays a role in inflammation. Its serum concentrations positively correlated with inflammatory cytokines, which are very high in septic shock and sepsis-related organ failure. Its main antibacterial mechanism is the regulation of iron metabolism, but it may also have an influence on the chemotaxis, adhesion, and migration of inflammatory cells. NGAL levels could be estimated in plasma, urine, and biological fluids such as peritoneal effluent, cerebrospinal fluid, saliva, tears, and human breast milk. Since plasma and urine NGAL concentrations increase rapidly after kidney injury, this could play a role as a biomarker in renal failure. Elevated NGAL concentrations were found in both acute and chronic kidney disease. In PD patients, NGAL could be a reliable marker of peritonitis. High serum NGAL concentrations were observed in atherosclerosis, hypertension, myocardial infarction, and heart failure. NGAL levels rose with the severity of atherosclerosis and coronary artery disease. Increased NGAL could also indicate the instability of atherosclerotic plaque. NGAL may be a predictor of the development of cardiorenal syndrome. NGAL also plays a role in metabolic complications. NGAL plasma levels have been found to be increased in obese individuals where it is likely to perform an anti-inflammatory function. The synthesis of NGAL is stimulated by increased serum glucose concentrations; therefore, NGAL levels are also elevated in diabetes and insulin resistance. NGAL is also involved in tumor processes such as cell proliferation, migration, multidrug resistance, and immunotolerance, which enhance the aggressiveness of the disease and promote metastases. Additionally, NGAL may possibly participate in the pathogenesis of primary Sjögren syndrome, cognitive impairment, and Alzheimer’s disease, as well as chronic obstructive pulmonary disease. However, this requires further study. The role of NGAL is summarized in Figure 4.

There is a lot of research on the role of NGAL in a variety of pathological situations. The knowledge of the different functions of this protein has been extended. Its role as a biomarker is promising; however, NGAL may also be a target for therapeutic procedures. Although numerous studies have been conducted, the exact mechanisms behind various activities of NGAL have not yet been completely understood.

## Figures and Tables

**Table 1:** The causes of increased NGAL in acute kidney injury.

Acute Kidney Injury	
Urine NGAL	Serum NGAL
The damage of proximal tubule and the impaired NGAL uptake Increased NGAL synthesis in distal part of nephron	The damage of glomerulus, impaired NGAL filtration and plasma NGAL accumulation Increased NGAL synthesis by neutrophils

NGAL, Neutrophil gelatinase-associated lipocalin.

## Data Availability

No new data were created or analyzed in this study. Data sharing is not applicable to this article.
